# Compound heterozygous mutation of the *SNX14* gene causes autosomal recessive spinocerebellar ataxia 20

**DOI:** 10.3389/fgene.2024.1379366

**Published:** 2024-04-09

**Authors:** Yuqi Shao, Saisai Yang, Jiafu Li, Lin Cheng, Jiawei Kang, Juan Liu, Jianhong Ma, Jie Duan, Yuanzhen Zhang

**Affiliations:** ^1^ Department of Obstetrics, Zhongnan Hospital of Wuhan University, Wuhan, China; ^2^ Hubei Clinical Research Center for Prenatal Diagnosis and Birth Health, Wuhan, China

**Keywords:** whole exome sequencing, growth and developmental delay, *SNX14*, genetic counseling, prenatal

## Abstract

**Objective:** The article aims to provide genetic counseling to a family with two children who were experiencing growth and developmental delays.

**Methods:** Clinical information of the proband was collected. Peripheral blood was collected from core family members to identify the initial reason for growth and developmental delays by whole exome sequencing (WES) and Sanger sequencing. To ascertain the consequences of the newly discovered variants, details of the variants detected were analyzed by bioinformatic tools. Furthermore, we performed *in vitro* experimentation targeting *SNX14* gene expression to confirm whether the variants could alter the expression of *SNX14*.

**Results:** The proband had prenatal ultrasound findings that included flattened frontal bones, increased interocular distance, widened bilateral cerebral sulci, and shortened long bones, which resulted in subsequent postnatal developmental delays. The older sister also displayed growth developmental delays and poor muscle tone. WES identified compound heterozygous variants of c.712A>T (p.Arg238Ter) and .2744A>T (p.Gln915Leu) in the *SNX14* gene in these two children. Both are novel missense variant that originates from the father and mother, respectively. Sanger sequencing confirmed this result. Following the guideline of the American College of Medical Genetics and Genomics (ACMG), the *SNX14* c.712A>T (p.Arg238Ter) variant was predicted to be pathogenic (P), while the *SNX14* c.2744A>T (p.Gln915Leu) variant was predicted to be a variant of uncertain significance (VUS). The structural analysis revealed that the c.2744A>T (p.Gln915Leu) variant may impact the stability of the *SNX14* protein. *In vitro* experiments demonstrated that both variants reduced *SNX14* expression.

**Conclusion:** The *SNX14* gene c.712A>T (p.Arg238Ter) and c.2744A>T (p.Gln915Leu) were identified as the genetic causes of growth and developmental delay in two affected children. This conclusion was based on the clinical presentations of the children, structural analysis of the mutant protein, and *in vitro* experimental validation. This discovery expands the range of *SNX14* gene variants and provides a foundation for genetic counseling and guidance for future pregnancies in the affected children’s families.

## Introduction

Cerebellar dysfunction may result in ataxia, a neurological disorder that causes a lack of coordinated muscle movement. Ataxia can be classified as acquired or hereditary (Schmahmann, 2004). Hereditary cerebellar ataxia is a genetically heterogeneous group of neurodegenerative diseases primarily characterized by progressive cerebellar ataxia. It accounts for 10%–15% of inherited neurological disorders and has varying prevalence rates among different regions and ethnicities (Klockgether and Paulson, 2011). Autosomal recessive cerebellar ataxias (ARCAs) are early-onset ataxias that are caused by progressive damage to the cerebellum or afferent nerve-related disorders and typically manifest before the age of 20 years. The prevalence of ARCAs is estimated to be three in one hundred thousand. ARCAs patients exhibit multisystemic phenotypes and often present with irregular gait, imbalance, dysphagia, movement disorders, and other motor-related features. These symptoms show extensive inter-and intra-familial variants (Ruano et al., 2014; Pan et al., 2021). The discovery of an increasing number of genes associated with ARCAs has been facilitated by the continuous advancement of molecular technologies. However, approximately 50% of ARCAs patients remain undiagnosed at the molecular level due to the emergence of new gene variants, as well as variants in regulatory regions, epigenetic regulation, gene dosage, or digenic inheritance patterns (Pfeffer et al., 2015; Minnerop et al., 2017). For affected families, it is particularly important to test for the exact causative variants of single-gene disorders and to understand the cause of the disease, which can provide an accurate diagnosis, personalized treatment and an effective follow-up strategy.

Recent studies reported that autosomal recessive spinocerebellar ataxia 20 (SCAR20) is caused by homozygous or compound heterozygous variants in *SNX14*. SCAR20 is characterized by early-onset cerebellar atrophy, intellectual developmental disorder, ataxia, autism, coarsened facial features, frequent hearing loss, and skeletal abnormalities. This study provided genetic counseling to a family of two clinically affected children who presented with ataxia. After genetic counseling, we carried out whole exome sequencing (WES). Structural analysis and *in vitro* experimental validation of variant loci were conducted to elucidate the genetic etiology, and the family was ultimately diagnosed with a *SNX14* compound heterozygous variant causing SCAR20.

## Materials and methods

### Subjects

Blood samples were obtained from two patients and their parents who were recruited for prenatal diagnosis at Zhongnan Hospital of Wuhan University. The study received approval from the Ethics Committee of Wuhan University Zhongnan Hospital and obtained written informed consent from the family.

### DNA extraction

Peripheral blood of the family was collected, and the whole genome DNA was extracted with a commercial kit (MyGenostics, China). DNA concentration was measured using Qubit 2.0 fluorometer (Thermo Fisher Scientific, USA).

### Whole exome sequencing (WES) analysis and bioinformatic prediction

The WES strategy was used to identify genetic variation. For DNA fragment preparation and hybridization capture, the Nextera DNA Flex Pre-enrichment Library Prep (Illumina, San Diego, USA), IDT’s Illumina Nextera DNA Unique Dual Indexes SetA, and Exome Panels-Enrichment Oligos Only (Illumina, San Diego, USA) were employed. The size of DNA fragments was detected using the Qsep100TM automatic nucleic acid protein analyzer. The DNA fragments underwent sequencing using the NovaSeq6000 platform (Illumina, USA) with 151bp double-end sequencing. The raw files were assessed in Fastq format and any data with less than 90% Q30 were excluded.

The sequencing readings were compared to the human reference genome (hg19/GRCh37) using the Burrows-Wheeler Aligner. PCR repeat fragments were removed using Picard V1.57. Variants were identified using Berry Genomics’ Verita Trekker^®^ Variants Detection System and GATK ANNOVAR and Enliven^®^ were then used to annotate and interpret the variants (Wang et al., 2010). The study analyzed patterns of single-gene inheritance, including autosomal dominant, autosomal recessive, and X-linked recessive. Additionally, the study examined mitochondrial DNA variation and possible imprinted gene variants. The harmful prediction of variants discovered using three different types of software, PolyPhen-2, SIFT, and Mutation Taster, was also evaluated. The variants underwent screening and were compared with the ClinVar database, HGMD, and other human genome databases to determine if they were reported as pathogenic. The pathogenicity of the mutant sites was annotated following the American College of Medical Genetics and Genomics (ACMG) criteria (Yang et al., 2019). Validation was performed using Sanger sequencing.

### Conservation analysis of SNX14 protein and re-modeling of mutated site

For conservation analysis, the protein sequences of SNX14 from human (*Homo sapiens*: NP_001990.2), mouse (*Mus musculus*: NP_034311.2), cattle (*Bos taurus*: NP_001265517.1), and zebrafish (*Danio rerio*: NP_001129262.1) were downloaded from NCBI; further, the sequences were inserted into MEGA11 and aligned using the MUSCLE algorithm (Tamura et al., 2021). For remodeling the structure of the mutated SNX14 protein, the amino acid sequence of SNX14 was first compared in SWISS-MODEL to obtain a template with a high degree of similarity. The predicted 3D structure of the SNX14 protein (AF-Q9Y5W7-F1) in the AlphaFoldDB database was highly fitted. Finally, the p.Gln915Leu variant was remodeled in PyMol based on the template downloaded.

### Cell culture

HEK293T cells (Procell, China) were cultured in Dulbecco’s modified Eagle’s medium (Hyclone, USA) with 10% fetal bovine serum (Procell, China) in an atmosphere of 5% CO_2_ at 37°C.

### Plasmids construction and transfection

The coding sequence (CDS) of *SNX14* (NM_153816.6) was cloned into the pcDNA3.1(+) vector (Invitrogen, USA) to generate *SNX14*-WT-HA. Based on *SNX14*-WT-HA, *SNX14*-c.712A>T-HA and *SNX14*-c.2744A>T-HA were generated. The constructs were verified by sequence. Lipofectamine 3000 (Thermo Fisher Scientific, USA) was selected for transfection, details were guided following the manufacturer’s protocol. 48 h after transfection, cells were harvested for the following steps.

### RNA extraction and quantitative polymerase chain reaction (qPCR)

Total RNA was extracted using a commercial kit (Aidlab, China), and the purity and amount of RNA were assessed using NanoDrop 2000 (Thermo Fisher Scientific, USA). 1ng RNA was used for reverse transcription with HiScript II mix (Vazyme, China), and qPCR was performed with SYBR mix (Vazyme, China) according to the manufacturer’s protocol. *GAPDH* was selected as the reference gene. The primer sequences of *GAPDH* were 5′-CAT​CAT​CCC​TGC​CTC​TAC​TGG-3′ (forward) and 5′-GTG​GGT​GTC​GCT​GTT​GAA​GTC-3′ (reverse), and the primer sequences of *SNX14* were 5′-TCC​CCG​AAA​CCT​TGC​TGC-3′ (forward) and 5′-GGC​TCG​TGT​CCA​ACT​GCT-3′ (reverse). The 2^−ΔΔCt^ method was used to calculate relative expression.

### Western blot

Total proteins were extracted using RIPA lysis buffer (Beyotime, China) and the concentration was measured according to the instructions of the BCA protein quantification Kit (Beyotime, China). Protein samples were separated by 10% SDS-PAGE (Epizyme, China) and transferred to PVDF membranes (Invitrogen, USA). The membranes were blocked with 5% milk for approximately 1 h at room temperature and then incubated in primary antibodies at 4°C overnight. The primary antibodies used were anti-HA (1:1000, C29F4, CST, USA), anti-β-tubulin (1:2000, 10068-1-AP, Proteintech, China). On the second day, TBST-washed membranes were incubated with horseradish peroxidase (HRP)-conjugated secondary antibodies (1:10000, SA00001-2, Proteintech, China) for 1 h at room temperature. Finally, protein bands were visualized using an ECL kit (Vazyme, China).

### Statistical analysis

Data were calculated as ratios relative to the corresponding negative controls, presented as means ± SD, and were appropriately analyzed by unpaired *t*-test with GraphPad Prism (Version 9, California). *p*-values <0.05 were considered statistically significant.

## Results

### Clinical findings

**FIGURE 1:**
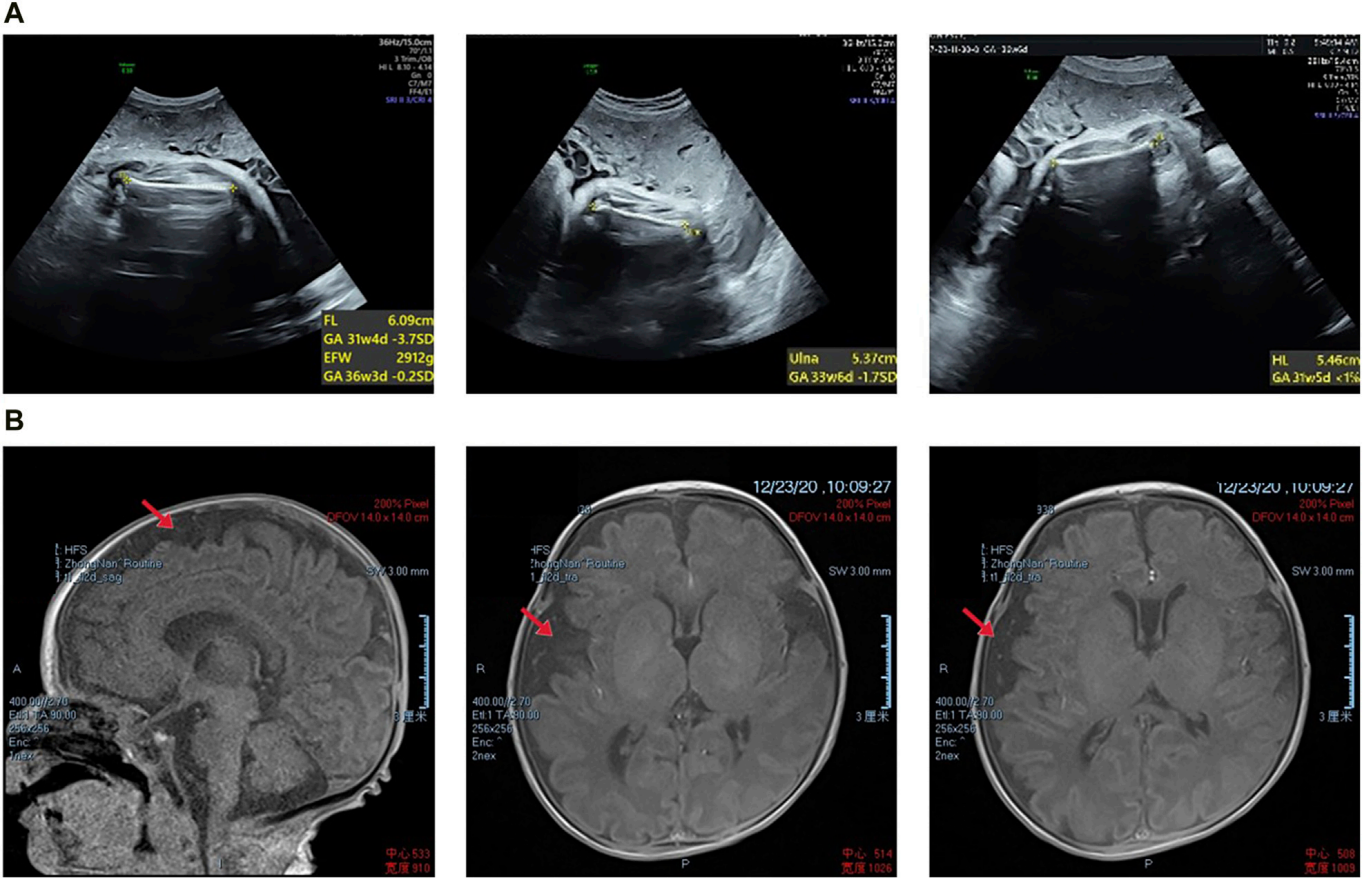
**(A)** Prenatal transabdominal ultrasound images were taken of the fetus at 31^+4^ weeks’ gestation, which showed short limbs. **(B)** The proband’s magnetic resonance imaging results revealed slight asymmetry in the bilateral brain parenchyma, wider gyri, and a slight widening of the subarachnoid space in the right frontal, parietal, and temporal lobes. Additionally, there was a slight leftward tilting of the midline structure.

The proband was diagnosed with a series of abnormalities during the prenatal period. The mother underwent amniocentesis, which revealed no significant chromosomal or gene copy number variants. The mid-term ultrasound examination indicated that the proband femur and humerus lengths were 2 standard deviations (SD) below the normal values, along with a slightly flattened frontal bone and increased interocular distance. A month later, magnetic resonance imaging (MRI) revealed bilateral enlargement of the extracerebral space in the fetal brain. A late-term systematic ultrasound examination showed that the measurements of the fetal long bones ranged from −1.7 to −4.1SD, suggesting possible fetal cartilage dysplasia (Figure 1A). Tracheal intubation was given to aid respiratory treatment, and continuous gastrointestinal decompression and intravenous nutritional support therapy were administered. This was followed by anti-infective, bleeding prevention, myocardial nutrition, and other symptomatic supportive therapies. A follow-up MRI revealed mild asymmetry in the brain parenchyma, widened brain gyri, and slight enlargement of the subarachnoid space in the right frontal, parietal, and temporal lobes, with a slight leftward shift of midline structures (Figure 1B). The infant was delivered by cesarean section at term pregnancy, after birth, the proband showed some severe symptoms such as dyspnea and three concave signs, with arterial oxygen saturation of 89%. At the age of 3, the proband exhibited poor language skills, only understanding simple commands, and was unable to walk independently.

During the consultation, it was discovered that the proband had an older sister who experienced overall growth delay, with a large head circumference at birth and the ability to walk with assistance at the age of 8. Both affected children exhibited broad foreheads, wide nasal bridges, long eustachian tubes, thick vermilion of the lower lip, and a small and narrow chin (Table 1).

**TABLE 1:** Clinical features of patients in this study and the literature. (See Supplementary Table S1 for detailed clinical information).

Clinical features	The proband	Older sister	Percent of patients displaying the feature	
Gender	M	F	M (18)	F (18)
Diagnosis age	3.5y	8y	−	−
Craniofacial Features	+	+	18/18	18/18
MRI(cerebellar atrophy)	+	NA	17/18	16/16
Neurodevelopment				
Intellectual disability	+	+	18/18	18/18
Delayed or absent language	+	+	17/17	18/18
Hypertonia	−	−	2/18	3/18
Hypotonia	+	+	14/18	14/18
Delayed fine/gross motor	−	+	17/17	18/18
Ataxia	+	+	11/16	14/18
Others				
Hearing loss	−	−	5/18	8/18
Elbow motion limitation	−	−	4/18	7/17
Scoliosis/kyphosis/clinodactyly/Talipes equino-varum	−	+	8/17	11/16
Walking with help (age, months)	−	7y	−	−

+, positive, represents the appearance of the phenotype.

−, negative, no obvious abnormal phenotype was observed.

F, female; M, male.

NA, not applicable.

### Variants analysis

**FIGURE 2:**
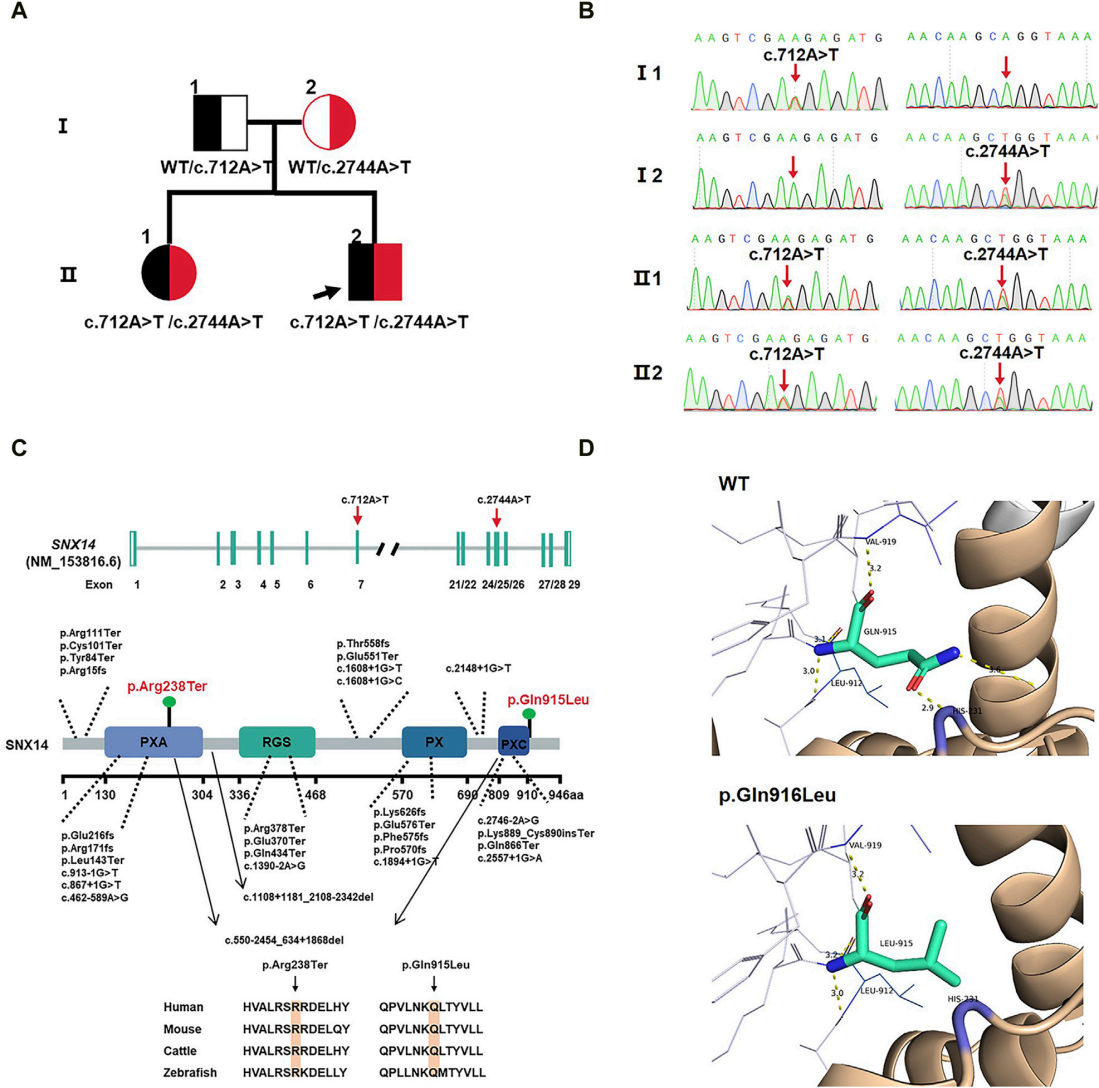
**(A)** Family pedigree. **(B)** Sanger sequencing in the proband and II:1 showing compound variants c.712A>T(p.Arg238Ter) and c.2744A>T(p.Gln915Leu) in *SNX14*, heterozygous in mother and father. **(C)** *SNX14* exons location of variants indicated. The structural model of the SNX14 protein and the position of the two variants identified in this study are shown in blue. Phylogenetic comparison of protein encoded by *SNX14* across species. **(D)** Homology modeling of wild-type and mutant *SNX14* c.2744A>T(p.Gln915Leu) variants. The remodeled structure revealed that the variant from Gln to Leu at amino acid 915 broke two hydrogen bonds, which could affect the stability of the SNX14 protein.

Based on OMIM and HGMD, this study successfully identified the proband and his older sister carrying a compound heterozygous variants of the *SNX14* gene consisting of c.712A>T (p.Arg238Ter) and c.2744A>T (p.Gln915Leu) by WES, which were respectively originated from the father and mother with normal phenotypes (Figures 2A, B). These variants were confirmed by Sanger sequencing. These missense variants were not present in the HGMD, the single nucleotide polymorphism (SNP) database (dbSNP), the 1000 Genomes Project (TGP) database, or the ClinVar database (Table 2). Furthermore, to our knowledge, these variants have not been described in the universal mutation database *SNX14* or reported in any published literature. Based on the ACMG guidelines, the c.712A>T (p.Arg238Ter) variant of *SNX14* was predicted to be pathogenic (P) (PVS1 + PM2_Supporting + PP3), and the c.2744A>T (p.Gln915Leu) variant of *SNX14* was predicted to be a variant of uncertain significance (VUS) (PM2_Supporting + PP3).

**TABLE 2:** *SNX14* variants observed in this family.

cDNA change	Protein change	Genomic position on chr6 (bp)	Variants type	SIFT^a^	PolyPhen2^a^	Mutation Taster^a^	REVEL^a^	GnomAD^b^	GERP++^c^	ACMG classification
NM_153816.6: c.712A>T	NP_722523.1: p.Arg238Ter	86259520	Nonsense	NA	NA	NA	NA	NA	5.39	pathogenic
NM_153816.6: c.2744A>T	NP_722523.1: p.Gln915Leu	86217687	Missense	Pathogenic	Pathogenic	NA	0.565	NA	5.06	VUS

*SNX14*: sorting nexin 14.

^a^ Variants assessment by SIFT, PolyPhen2, Mutation Taster, and REVEL. NA is not available.

^b^ Frequency in unselected individuals in the GnomAD, database.

^c^ Nucleic acid conservative prediction by GERP.

### Protein modeling

The two mutated sites of the SNX14 protein were highly conserved in four species (Figure 2C). We constructed the 3D structure of the p.Gln915Leu variant (Figure 2D). The remodeled structure revealed that the variant from Gln to Leu at amino acid 915 broke two hydrogen bonds, which could affect the stability of the SNX14 protein.

### The expression of SNX14

**FIGURE 3:**
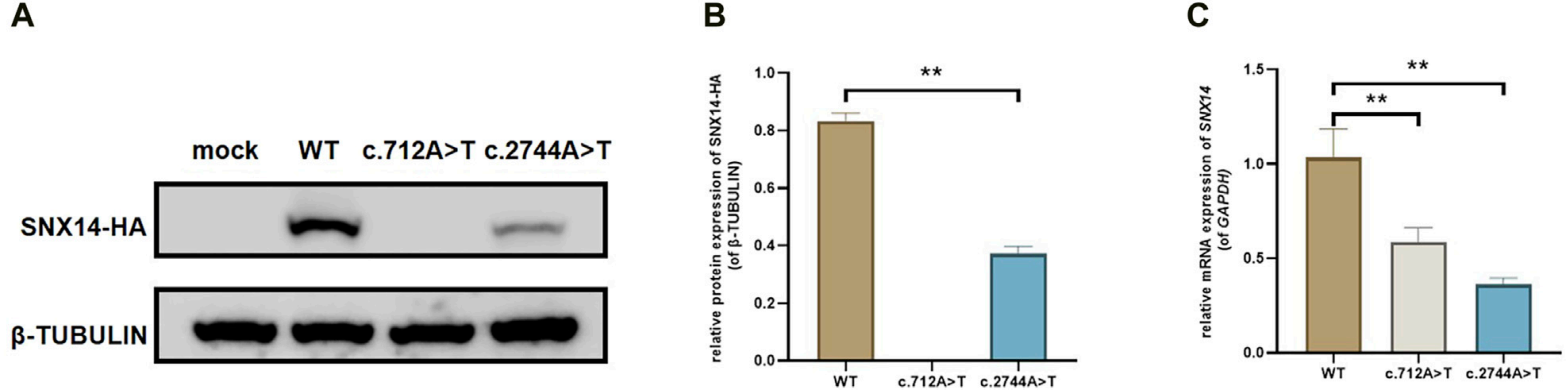
The effect of the variant on SNX14 expression. **(A, B)** Western blot staining of wild-type and splice site mutant highlighting the relative protein amount. **(C)** Relative mRNA levels of SNX14 mutant and wild type. The values were the Mean ± SD. *p < 0.05, **p < 0.01, (*n* = 3).

We constructed C-termination HA-tagged plasmids, as shown in Figure 3, c.712A>T (p.Arg238Ter) variant led to early termination of protein translation with no band in the related lane, meanwhile, we found that the mRNA expression of *SNX14* was decreased with the mutation of c.712A>T. We surprisingly found that c.2744A>T (p.Gln915Leu) led to the deletion of SNX14 protein expression which was also observed at the mRNA level. Taken together, *SNX14* c.712A>T (p.Arg238Ter) and c.2744A>T (p.Gln915Leu) were reclassified as P (PVS1 + PM2_Supporting + PP1 + PP3 + PP4) and VUS (PM2_Supporting + PP1 + PP3 + PP4) by ACMG guidelines (Supplementary Table S2).

## Discussion

SCAR20 is an early-onset form of ARCAs that was recently reported. It typically occurs in early childhood, although rare cases have been diagnosed during infancy or fetal development (Thomas et al., 2014). The main features of SCAR20 include progressive cerebellar atrophy, ataxia, and severe intellectual disability. Some patients may also exhibit craniofacial abnormalities, such as macrocephaly, a prominent forehead, and thick lips. In addition, patients may exhibit symptoms such as epilepsy, deafness, and skeletal abnormalities (Thomas et al., 2014; Akizu et al., 2015). The primary phenotype of the fetus can only be observed postnatally, and prenatal ultrasound examinations often fail to detect these features. Furthermore, many of the characteristic phenotypes of affected individuals may not be present during the prenatal stage, such as intellectual disability in the absence of gross brain structural anomalies. Currently, there is no description of intrauterine phenotypes associated with SCAR20. This study retrospectively reviewed the prenatal examinations of the proband. The mid-term ultrasound examination showed fetal femur and humerus lengths below -2SD, along with a slightly flattened forehead and increased interocular distance. A month later, a fetal MRI revealed bilateral enlargement of the brain’s extracerebral space. The late-term systematic ultrasound examination revealed that the measurements of the fetal long bones ranged from −1.7SD to −4.1SD. This study contributes to the intrauterine phenotype of SCAR20, highlighting the importance of detailed genetic counseling in cases where prenatal ultrasound suggests fetal limb shortening and the possibility of SCAR20 should be excluded. Both the proband and his older sister in the present study have craniofacial features that are predominantly characterized by broad foreheads, wide nasal bridges, long eustachian tubes, thick vermilion of the lower lip, and a small and narrow chin, and the same facial features were present in all the patients reported (Akizu et al., 2015). In addition, the two affected children in this study and the majority of patients reported in the literature were associated with neurodevelopmental delays such as intellectual disability, delayed or absent language, hypertonia or hypotonia, and ataxia, and MRIs would have shown cerebellar atrophy in the majority of patients, providing imaging support for clinicians (Levchenko et al., 2023). Nevertheless, it should be noted that these clinical signs are progressive and absent in early infancy, making clinical recognition in infants challenging. Thus, there is a greater need to focus on the interrogation of family history and the use of molecular testing techniques. In addition, hearing loss and abnormal skeletal development are also found in patients with SCAR20, but not all patients have these features (Thomas et al., 2014); for example, the proband and his older sister in this study have normal hearing and no significant abnormalities in skeletal development (Table 1; Supplementary Table S1).

Loss-of-function variants in *SNX14* cause SCAR20. SNX14 is a member of the sorting nexin (SNX) family, specifically the PXA-RGS-PX-PXC subclass of SNX proteins (Zhang et al., 2021). Research has shown that a higher proportion of patients with early-onset cerebellar atrophy and ataxia carry variants in *SNX14* (9.88%) compared to other known pathogenic genes such as *GRID2* (2.47%), *NPC1* (1.23%), and *SETX* (1.23%) (Akizu et al., 2015). The majority of these variants are loss-of-function variants occurring primarily in the PX and RGS domains. To date, 30 different pathogenic and potentially pathogenic variants, including nonsense variants, exon deletions, and splice site variants, have been reported in data from 47 patients from 25 families (Maia et al., 2020). Studies on animals have shown that the SNX14 protein is highly expressed in the mouse brain. It gradually increases during neuronal development and maturation and plays a critical role in maintaining normal excitability and synaptic transmission in neurons (Zhang et al., 2021). Knockout mouse models of *Snx14* have shown severe motor impairments and cell-autonomous Purkinje cell degeneration, revealing the pathogenic mechanism of cerebellar ataxia (Huang et al., 2014). This study found that the proband and his older sister carried compound heterozygous variants in the *SNX14* gene, c.712A>T (p.Arg238Ter) and c.2744A>T (p.Gln915Leu), respectively. These variants were inherited from phenotypically normal parents. According to the ACMG guidelines, the *SNX14* c.712A>T (p.Arg238Ter) variant was classified as P (PVS1 + PM2_Supporting + PP3), while the *SNX14* c.2744A>T (p.Gln915Leu) variant was classified as VUS (PM2_Supporting + PP3).

The missense variant c.712A>T (p.Arg238Ter) is located in the PXA structural domain of the SNX14 protein. This variant resulted in the premature termination of protein translation (Bryant et al., 2018). *In vitro* experiments showed decreased expression of SNX14 protein and mRNA levels. SNX14 is an ER-anchored integral membrane protein, and the PXA structural domain is responsible for SNX14 binding to organelle membranes. It is hypothesized that the c.712A>T variant might affect cellular lipid transport, and excess lipids in neuronal cells disrupt the balance of lipid metabolism, which may affect mitochondrial function. On the other hand, the c.2744A>T (p.Gln915Leu) variant is located in none of the important structural domains of the SNX14 protein. The structure of this mutation was remodeled, and it was found that the mutation altered the hydrogen bonding between the amino groups, which may reduce the stability of SNX14. *In vitro* experiments demonstrated that the expression of SNX14 protein and mRNA was downregulated. Both variants decreased SNX14 expression, and insufficient levels of SNX14 *in vivo* could result in SCAR20 which may be the result of neuronal lipotoxicity and mitochondrial dysfunction. However, *SNX14* c.2744A>T (p.Gln915Leu) remains classified as VUS (PM2_Supporting+PP1+PP3+PP4) following ACMG guidelines. The present study has identified two variants in *SNX14*, c.712A>T (p.Arg238Ter) and c.2744A>T (p.Gln915Leu), as the genetic cause of SCAR20 in the family. However, as the c.2744A>T is a VUS variant, it is necessary to further study the role of this variant in regulating protein structure and function by *in vivo* experiments. This will not only expand the spectrum of *SNX14* gene variants and the prenatal clinical phenotype of SCAR20 but also guide prenatal diagnosis and clinical genetic consultation.

Currently, ARCAs classification is based on pathogenic genes, pathogenesis, and associated signaling pathways, which include mitochondrial metabolism-related, DNA repair/genome homeostasis-related, and lipid metabolism-related. This classification is useful for treating these diseases. If a treatment is effective for a certain disease, it is expected to have the same effect on other diseases with the same molecular mechanism. However, ARCAs have a multi-systemic phenotype, often with extensive inter- and intra-familial variation. SCAR20 is a disease that is challenging to distinguish from other ARCAs in terms of clinical presentation. The development and advancement of molecular diagnostic techniques have made it possible to definitively diagnose SCAR20 using WES. This is essential for informing parents about their pregnancy options and management, as well as providing more accurate genetic diagnostics of the risk of recurrence in future pregnancies and prior to implantation. Additionally, it allows for more precise genetic counseling for preimplantation genetic diagnosis.

## Conclusion

This study reports the identification of the c.712A>T (p.Arg238Ter) and c.2744A>T (p.Gln915Leu) variants in a Chinese family for the first time. Bioinformatics and *in vitro* experiments have demonstrated that these variants impact the structure and expression of the SNX14 protein, resulting in SCAR20. This expands the spectrum of variants in the *SNX14* gene. SCAR20 is rare during the fetal period and childhood and can be difficult to distinguish from other ARCAs. The presentation during the fetal period may be atypical, and the etiology of the fetal abnormalities of the limbs, long bones, and/or facial development should be emphasized. Attention should also be given to the cause of fetal short limbs and/or facial developmental abnormalities. It is important to inquire about the family history of the disease and improve genetic testing to clarify the genetic cause. This will provide a theoretical basis for pregnancy management and guidance for future childbearing.

## Data Availability

The datasets presented in this study can be found in online repositories. The data presented in the study are deposited in the GenBank repository, accession numbers are SAMN40659395.

## Supplementary material

SUPPLEMENTARY FIGURE S1 Uncut western blot figure.
