# Supplementary figures and images for: Compound heterozygous mutation of the SNX14 gene causes autosomal recessive spinocerebellar ataxia 20
Source: Front Genet. 2024 Apr 9;15:1379366. doi: 10.3389/fgene.2024.1379366 (PMC11035801; doi:10.3389/fgene.2024.1379366)

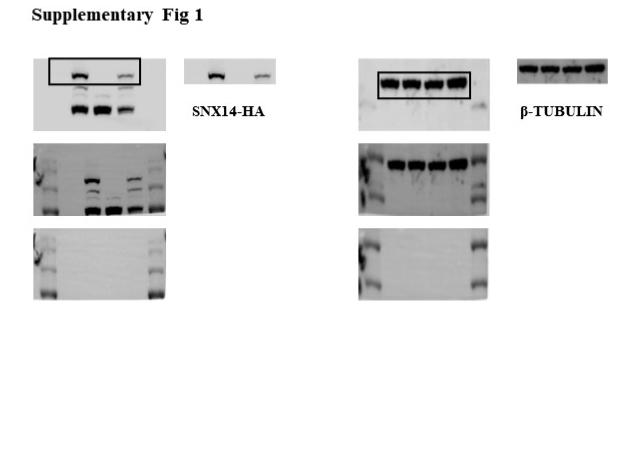

Supplement: Supplementary file 2 [file Image1.JPEG]
