# Supplementary material for: Compound heterozygous mutation of the SNX14 gene causes autosomal recessive spinocerebellar ataxia 20
Source: Front Genet. 2024 Apr 9;15:1379366. doi: 10.3389/fgene.2024.1379366 (PMC11035801; doi:10.3389/fgene.2024.1379366)
Supplement: Supplementary file 3 [file Table4.DOCX]

| ACMG/AMP criteria | c.712A>T | c.2744A>T |
| --- | --- | --- |
| PVS1 | c.712A>T variant led to early termination of protein translation | - |
| PM2_Supporting | These missense variants were not present in the HGMD, the single nucleotide polymorphism (SNP) database (dbSNP), the 1000 Genomes Project (TGP) database or the ClinVar database | |
| PP3 | Multiple statistical methods predicted that the variant would have a deleterious effect on the gene or gene product(Variants assessment by SIFT, PolyPhen2, and Mutation Taster). | |
| PP1 | This variant was detected in the proband and his older sister of this family line. | |
| PP4 | The proband and his older sister combined with the genetic test results were highly consistent with SCAR20. | |

**Supplementary Table 2**: Applied ACMG/AMP criteria.
